# Risk Factors for Intrapartum Cesarean Section Delivery in Low-risk Multiparous Women Following at Least a Prior Vaginal Birth (Robson Classification 3 and 4)

**DOI:** 10.1055/s-0041-1731378

**Published:** 2021-07-27

**Authors:** Gul Nihal Buyuk, Hatice Kansu-Celik, Zeynep Asli Oskovi Kaplan, Burcu Kisa, Sule Ozel, Yaprak Engin-Ustun

**Affiliations:** 1 Department of Obstetrics and Gynecology, University of Health Sciences, Zekai Tahir Burak Woman's Health, Education and Research Hospital, Ankara, Turkey

**Keywords:** cesarean section, vaginal delivery, risk factors, fetal abdominal circumference, cervical dilatation

## Abstract

**Objective** The aim of the present study was to evaluate the risk factors for cesarean section (C-section) in low-risk multiparous women with a history of vaginal birth.

**Methods** The present retrospective study included low-risk multiparous women with a history of vaginal birth who gave birth at between 37 and 42 gestational weeks. The subjects were divided into 2 groups according to the mode of delivery, as C-section Group and vaginal delivery Group. Risk factors for C-section such as demographic characteristics, ultrasonographic measurements, smoking, weight gain during pregnancy (WGDP), interval time between prior birth, history of macrosomic birth, and cervical dilatation at the admission to the hospital were obtained from the charts of the patients. Obstetric and neonatal outcomes were compared between groups.

**Results** The most common C-section indications were fetal distress and macrosomia (33.9% [ *n* = 77 and 20.7% [ *n* = 47] respectively). A bivariate correlation analysis demonstrated that mothers aged > 30 years old (odds ratio [OR]: 2.09; 95% confidence interval [CI]: 1.30–3.34; *p* = 0.002), parity >1 (OR: 1.81; 95%CI: 1.18–2.71; *p* = 0.006), fetal abdominal circumference (FAC) measurement > 360 mm (OR: 34.20; 95%CI: 8.04—145.56; *p* < 0.001)) and < 345 mm (OR: 3.06; 95%CI: 1.88–5; *p* < 0.001), presence of large for gestational age (LGA) fetus (OR: 5.09; 95%CI: 1.35–19.21; *p* = 0.016), premature rupture of membranes (PROM) (OR: 1.52; 95%CI: 1–2.33; *p* = 0.041), and cervical dilatation < 5cm at admission (OR: 2.12; 95%CI: 1.34–3.34; *p* = 0.001) were associated with the group requiring a C-section.

**Conclusion** This is the first study evaluating the risk factors for C-section in low-risk multiparous women with a history of vaginal birth according to the Robson classification 3 and 4. Fetal distress and suspected fetal macrosomia constituted most of the C-section indications.

## Introduction

Increasing cesarean section (C-section) rates are becoming a concern especially in countries with higher C-section rates. The C-section rates have increased gradually in recent years, especially in middle- and high-income countries, without any increase in indications or strict medical reasons. 1 Cesarean section rates > 15% are not recommended by the World Health Organization (WHO). 2 In Turkey, the C-section rate among all deliveries has increased from 21% in 2012 to 53% in 2015. 3 Various reasons for why mothers and obstetricians prefer C-section have been postulated for this increase, including prior C-section deliveries, advanced maternal age, systemic diseases such as hypertension and diabetes mellitus, multiple pregnancies, fetal distress, macrosomic fetus, malpresentation of fetus, cephalopelvic disproportion, prolonged labor, and insufficient supplementary health network. 4

In recent years, the rate of incidences requiring a C-section is steadily increasing all over the world. Advanced maternal age, chronic health problems, multiple pregnancies as a result of the development of assisted reproductive technologies, and an insufficient supplementary health network can be considered as the reasons why mothers and obstetricians prefer a C-section. 5 Notwithstanding, C-section includes some short and long-term risks, such as reduction in fertility, increased risk of maternal mortality and morbidity, poor obstetric outcomes, requirement of treatment in an intensive care unit (ICU), and need for blood transfusion due to the risky surgical procedure. Women who delivered vaginally are much more likely to have a subsequent vaginal birth. 6 Determination of the risk factors that we can change in multiparous women for subsequent C-section may help to reduce unintended primary C-sections. There are previous reports on risk factors of intrapartum C-sections in multiparous women in limited patient groups; however, a detailed analysis including a large cohort have not been reported according to our knowledge. So, we aimed to demonstrate the risk factors for intrapartum C-section in low-risk women with a history of vaginal birth.

## Methods

The present retrospective case-control study included low-risk multiparous women with a history of at least 1 prior vaginal birth who gave birth at between 37 and 42 gestational weeks in the University of Health Sciences, Zekai Tahir Burak Woman's Health and Research Hospital, between January 2017 and July 2017. The project was approved by the Institutional Review Board of the hospital (No: 41/2018, February 2018). The women were divided into 2 groups according to the mode of delivery, as Cesarean section (C-section) Group and vaginal delivery Group (Control group). Deliveries were included in the study according to the Robson classification 3 and 4. 6 Women who underwent intrapartum C-section with a history of vaginal birth were enrolled into the study group. The control group was randomly constituted by women who gave birth vaginally. Demographic characteristics, parity, ultrasonographic measurements including estimated fetal weight (EFW), biparietal diameter (BPD), fetal abdominal circumference (FAC), smoking, weight gain during pregnancy (WGDP), interval time between prior birth, history of macrosomic birth, cervical dilatation at the admission to the hospital, obstetric and neonatal outcomes were obtained from the charts and electronic database of the patients. The exclusion criteria included multiple gestation, nonmedical oxytocin induction, previous uterine scarring, maternal fever, gestational diabetes, pregnancy-induced hypertension, oligohydramnios, and any history of chronic systemic disease.

Gestational age was determined by the reported last menstrual period and dating of first-trimester ultrasound measurements. The body mass index (BMI) was calculated as weight divided by height in m ^2^ . Premature rupture of membranes (PROM) was defined as the rupture of membranes before the onset of labor. 7 Infants were classified by gestational age and birthweight into small-for-gestational-age (SGA), appropriate for gestational age (AGA), and large-for gestational age (LGA) categories. 8 Ultrasonography was performed on all patients within ∼ 24 hours before delivery to assess the presentation of fetuses, EFW, BPD, FAC, placental site, and amniotic fluid volume. The FAC was measured at the level where the umbilical vein passes through the liver. The BPD was measured as a transverse image of the head with the cursors placed from the leading edge to leading edge of the skull bones. Formulas have been calculated to estimate the fetal weight using combinations of BPD, HC, FL, and AC. The Hadlock formula was used for EFW. 8 According to the Bishop score, oxytocin infusion or in the presence of an unfavorable cervix, a vaginal insert containing 10 mg timed-release dinoprostone (PGE2) was used in cases of medical indications such as ineffective contractions accompanying cervical dilatation and effacement, decreased fetal movements, nonreassuring fetal heart rate, prolonged PROM, and/or post-term pregnancy. 9

Statistical analyses were performed using SPSS Statistics for Windows, version 17 (SPSS Inc., Chicago, IL, USA). The distribution of the parameters was analyzed by the Kolmogorov-Smirnov and Shapiro-Wilk tests. The continuous variables with normal distribution were presented by means ± standard deviation (SD) and were compared by the independent samples *t* -test. Nonparametric variables without normal distribution were tested by the Mann-Whitney U test. The chi-squared and the Fisher exact tests were used for categorical data.

For the multivariate analysis, possible risk factors identified in the univariate analyses were further entered into the binary logistic regression analysis to determine independent predictors of C-section. The significance boundary was set at 0.05. In the post-hoc power analysis, the power of the study was found to be between 0.80 and 1 (for age, FAC, cervical dilatation at admission, SGA, PROM, and LGA, the power of the study was 0.80. 0.80, 0.80, 0.84, 0.91, and 1, respectively), with a 0.5 effect size and a 0.05 error rate for 500 participants consisting of 227 subjects in the C-section group and 273 subjects in the Control group (Newton.stat.ubc.ca).

## Results

During the study period, 2,268 healthy low-risk multiparous women with a history of vaginal birth who met the inclusion criteria at between 37 and 42 gestational weeks gave birth in our hospital. Of these, 10% of the patients ( *n* = 227) had given birth through a C-section. The control group ( *n* = 228) was chosen randomly from women giving birth by the vaginal route in the same cohort. The age of the mothers, parity, gestational age at delivery, rate of post-term pregnancy, BMI, birthweight, WGDP, and macrosomia were significantly higher in the C-section Group. The rate of a history of macrosomic birth was higher for the Control group, and the difference was statistically significant: 34 (12%) versus 15 (6%); *p* = 0.020). The incidence of newborns with Apgar 1 ^st^ minute score < 7 was significantly higher in the C-section group ( *p* = 0.006). Also, the rate of neonatal intensive care unit (NICU) admission was significantly higher in the C-section group(7 [2.6%] versus 15 [6.6%]; *p* = 0.048)]. There were no other significant differences between the groups. Demographic, obstetrics and neonatal characteristics are listed in Table 1 . Table 1 also shows the ultrasonographic and labor characteristics of the two groups. The EFW, rate of EFW ≥ 4,000 g, FAC, and rate of PROM were significantly higher in the C-section Group. The cervical dilatation at admission, the requirement of induction, and meconium-stained amnion were higher in the Control group, with a statistically significant difference. The C-section indications were fetal distress (33.9%; *n* = 77), macrosomia (20.7%; *n* = 47), cephalopelvic disproportion (16.3%; *n* = 37), malpresentation (14.5%; *n* = 33), failure to progress in labor (12.3%; *n* = 28), and others (2.2%; *n* = 5).

**Table 1:** Demographic, obstetrics and neonatal characteristics

Variable	Control group	C-Section group	*p-value*
	( *n* = 273)	( *n* = 227)	
Age (years old) (mean ± SD)	30.24 ± 5.56	32.86 ± 6.38	< 0.001 *
Gravidity, median (min-max)	3 (2–7)	3(2–8)	0.051
Parity, median (min-max)	1.2 ± 1.1	2.3 ± 1.4	0.032 *
Abortion, median (min-max)	0 (0–2)	0 (0–2)	0.921
BMI (kg/m ^2^ ) (mean ± SD)	31.79 ± 3.73	32.59 ± 4.64	0.032 *
Gestational age at delivery (weeks) (mean ± SD)	39.06 ± 1.28	39.39 ± 1.25	0.004 *
Post-term pregnancy (> 41 weeks) (n, %)	35 (12%)	47 (20%)	0.021 *
Birthweight (g) (mean ± SD)	3333 ± 374	3561 ± 588	< 0.001 *
SGA, n (%)	14 (5%)	21 (12%)	0.019 *
LGA, n (%)	26 (10%)	57 (33%)	< 0.001 *
Birthweight > 4,000 g, median (min-max)	13 (5%)	52 (23%)	< 0.001 *
Apgar scores n (%)			
1 ^st^ minute < 7	5 (1.8%)	16 (7%)	0.006 *
5 ^th^ minute < 7	1 (0.4%)	3 (1.3%)	0.334
NICU (n, %)	7 (2.6%)	15 (6.6%)	0.048 *
Smoking n (%)	40 (14%)	46 (20%)	0.121
Birthweight of previous child (g) (mean ± SD)	3,448 ± 372	3,387 ± 361	0.065
History of macrosomic birth	34 (12%)	15 (6%)	0.020 *
Time interval between previous birth (years) Median (min-max)	4 (2–13)	4 (2–8)	0.735
WGDP (kg) median (min-max)	15 (10–24)	17 (8–28)	0.005 *
Estimated fetal weight (g) (mean ± SD)	3351 ± 333	3639 ± 558	< 0.001 *
Estimated fetal weight ≥ 4,000 g (n, %)	18 (6.6%)	65 (28.6%)	< 0.001 *
BPD (mm) median (min-max)	95 (86–105)	95 (82–100)	0.188
FAC (mm) median (min-max)	339 (228–371)	349 (318–385)	< 0.001 *
Cervical dilatation (cm) median (min-max)	4 (2–10)	3 (2–8)	< 0.001 *
Requirement of induction (n, %)	134 (35%)	66 (22%)	0.002 *
PROM (n, %)	83 (30%)	94 (45%)	0.011 *
Meconium stained amnions (n, %)	17 (6%)	5 (2%)	< 0.030 *

Abbreviations: BMI, body mass index; BPD, biparietal diameter; FAC, fetal abdominal circumference; LGA, Large for gestational age; NICU, requirement of neonatal intensive care unit; PROM, premature rupture of membranes; SGA, small for gestational age; WGDP, weight gain during pregnancy.

* *p* < 0.05, significant.

Table 2 shows the results of the binary logistic regression analysis. A bivariate correlation analysis demonstrated that mothers aged > 30 years old (odds ratio [OR]: 2.09; 95% confidence interval [CI]: 1.30–3.34; *p* = 0.002), parity >1 (OR: 1.81; 95%CI: 1.18–2.71; *p* = 0.006), fetal abdominal circumference (FAC) measurement > 360 mm (OR: 34.20; 95%CI: 8.04—145.56; *p* < 0.001)) and < 345 mm (OR: 3.06; 95%CI: 1.88–5; *p* < 0.001), presence of large for gestational age (LGA) fetus (OR: 5.09; 95%CI: 1.35–19.21; *p* = 0.016), premature rupture of membranes (PROM) (OR: 1.52; 95%CI: 1–2.33; *p* = 0.041), and cervical dilatation < 5cm at admission (OR: 2.12; 95%CI: 1.34–3.34; *p* = 0.001) were associated with the group requiring a C-section.

**Table 2:** Result of binary logistic regression analysis for risk of C-section

Variable	Wald	OR (95%CI)	*p-value*
Age > 30 years old	9.522	2.09 (1.30–3.34)	0.002 *
Parity > 1	7.408	1.81 (1.18–2.71)	0.006 *
BMI > 30 kg/m ^2^	0.693	1.19 (0.76–1.86)	0.437
LGA	5.794	5.09 (1.35–19.21)	0.016 *
SGA	9.641	0.32 (0.15–0.65)	0.002 *
PROM	3.833	1.52 (1–2.33)	0.041 *
EFW ≥ 4,000 g	0.004	1.04 (1.27–3.89)	0.951
FAC > 360 mm	22.859	34.20 (8.04—145.56)	< 0.001 *
FAC < 345 mm	20.172	3.06 (1.88–5)	< 0.001 *
Post-term pregnancy (> 41 weeks)	0.509	0.80 (0.44–1.45)	0.475
WGDP >15 kg	2.711	0.70 (0.46–1.07)	0.100
Cervical dilatation < 5 cm	10.525	2.12 (1.34–3.34)	0.001 *

Abbreviations: BMI, body mass index; BPD, biparietal diameter; EFW, estimated fetal weight; FAC, fetal abdominal circumference; LGA, large for gestational age; PROM, premature rupture of membrane; SGA, small for gestational age; WGDP, weight gain during pregnancy.

* *p* < 0.05, significant.

## Discussion

In the present study, we evaluated the risk factors for C-section in low-risk women with a history of at least one prior vaginal birth. Previous studies demonstrated that the demand for a C-section was associated with a fear of childbirth, previous C-Section, and unfavorable delivery experience. 9 The decision to perform a C-section depends, at least in part, on the presence of several evolving conditions, such as pre-eclampsia, premature PROM, fetal growth restriction, and maternal chronic medical condition in multiparous women. 10 We excluded these parameters in our study. Besides, nowadays, women are older when they give birth, and their BMIs have increased. 11 Ennen et al. 12 showed that advanced maternal age and high BMI increased the possibility of C-section. In addition, the increase in the number of gravidity and parity increases the likelihood of many adverse pregnancy outcomes. In a population-based analysis using an Italian region data including Robson classification 3 and 4, the authors found that increased maternal age was an independent risk factors for C-section. 13 We demonstrated that increased mother's age, gravidity, parity, and BMI were significantly higher in the C-section Group.

In our study, less cervical dilatation at admission was another important risk factor for C-section in multiparous women. Some authors suggested that the increased C-section rate was associated with unfavorable cervix but unaffected by labor induction. 14 Some studies proposed a decrease in C-section delivery with admission at higher cervical dilatation. 15 16 Recent studies showed that the active phase of labor may not start until 6 cm of cervical dilatation; this is consistent with the results of our study. 17 Some retrospective studies have demonstrated the relationship between cervical dilatation upon admission and C-section rates. Holmes et al. 18 showed that C-section rates were significantly higher in women who were admitted with between 0 and 3 cm of cervical dilatation when compared with women who were admitted with between 4 and 10 cm of cervical dilatation among multiparous women (5.7 versus 1.3%; OR: 4.73; 95%CI: 2.64–8.49). Bailit et al. 19 demonstrated that cervical examination with ≤ 4 cm dilatation at admission was associated with significantly increased C-section rates in multiparous women (3.1 versus 1.4%; *p* < 0.001). Recently, a prospective cohort study by Wood et al. 20 found that, especially in multiparous women, lower cervical dilatation at admission was a modifiable risk factor for C-section. Similar to previous studies, we found that women with cervical dilatation < 5 cm at admission were 2 times more likely to undergo a C-section. Fetal distress has been shown to contribute to increase C-section rates. With results similar to ours, Çelik et al. 21 conducted a study in Turkey showing that fetal distress was the most common C-section indication in multiparous women. Intrapartum hypoxia is a condition linked between maternal and neonatal morbidity. Uterine contractions during labor are associated with a reduction in uterine blood flow by up to 60%, which may lead to fetal decompensation, particularly in the presence of inadequate placental function. 22 We found that FAC < 345 mm and PROM were dependent risk factors for C-section delivery after vaginal birth. Also, newborns with 1 ^st^ minute Apgar score < 7 and requirement of admission to the NICU were higher in the C-section group, and this was consistent with our findings. These conditions may predispose to intrapartum hypoxia, which is clinically associated with fetal heart rate abnormalities.

Fetal macrosomia has potentially serious effects that may result in a traumatic birth for newborns and mothers. Although the cause is unknown in many LGA cases, these factors associated with this condition include maternal diabetes, history of macrosomic delivery, multiparity, prepregnancy maternal obesity, excessive WGDP, and post-term pregnancy. 23 Weiner et al. 24 found that the rate of C-section in fetuses estimated ultrasonographically as weighing ≥ 4,000 g was 2 times higher than in controls (50.7 versus 24.9%; *p* < 0.05)(37). Some authors support that adverse outcomes such as hemorrhage, shoulder dystocia, brachial plexus injury, and asphyxia during vaginal delivery caused by macrosomia can be prevented by elective C-section or early induction of labor. 24 Also, medicolegal problems that may occur as a result of complications after vaginal delivery may play a role in the preference by part of physicians for C-section. 24 In the regression analysis, we demonstrated that advanced maternal age, increased parity, WGPD, and FAC > 362 mm were a significant factor for C-section.

The main limitation of the present study is its retrospective design. To the best of our knowledge, this is the first study evaluating the risk factors for C-section in low-risk multiparous women with a history of vaginal birth according to Robson classification 3 and 4. A total of 10% of patients had given birth through a C-section. We found that increased maternal age, parity, presence of LGA fetus, FAC > 360 mm or < 345 mm, PROM, and decreased cervical dilatation at admission < 5 cm were significant risk factors for C-section delivery in low-risk multiparous women with history of prior vaginal birth. If we look at the indications, 55% of C-section indications were fetal distress and suspected fetal macrosomia in our study population. Especially low-risk multiparous women with PROM and unfavorable cervical dilatation at admission should be followed-up carefully for the risk of fetal distress. On the other hand, although antenatal suspected macrosomia is associated with a marked increase in C-sections, these cannot provide a significant reduction in the incidence of shoulder dystocia or of birth trauma. 25 Therefore, the management of suspected fetal macrosomia requires clear contact and decision-making between the woman and her physician. Although our study was retrospectively designed, the number of patients was quite sufficient. However, further randomized prospective research is needed for the management of labor in low-risk multiparous women with a history of vaginal birth.
